# Roles of E3 Ubiquitin-Ligases in Nuclear Protein Homeostasis during Plant Stress Responses

**DOI:** 10.3389/fpls.2018.00139

**Published:** 2018-02-08

**Authors:** Irene Serrano, Laura Campos, Susana Rivas

**Affiliations:** LIPM, Université de Toulouse, INRA, CNRS, Castanet-Tolosan, France

**Keywords:** E3 ubiquitin-ligase, plant cell nucleus, post-translational modification, 26S proteasome, transcription factor

## Abstract

Ubiquitination, the reversible protein conjugation with ubiquitin (Ub), is a post-translational modification that enables rapid and specific cellular responses to stimuli without requirement of *de novo* protein synthesis. Although ubiquitination also displays non-proteolytic functions, it often acts as a signal for selective protein degradation through the ubiquitin-proteasome system (UPS). In plants, it has become increasingly apparent that the UPS is a central regulator of many key cellular and physiological processes, including responses to biotic and abiotic stresses. In the nucleus, protein regulation *via* the UPS orchestrates gene expression, genome maintenance, and signal transduction. Here, we focus on E3 Ub-ligase proteins as major components of the ubiquitination cascade that confer specificity of substrate recognition. We provide an overview on how they contribute to nuclear proteome plasticity during plant responses to environmental stress signals.

## Introduction

Modulation of protein activity by post-translational modifications (PTMs) enables rapid and specific cellular responses to stimuli without the requirement of energy-consuming *de novo* protein synthesis. Ubiquitination regulates a plethora of cellular functions, from growth and development to responses to biotic and abiotic stimuli (Moon, 2004; Vierstra, 2009). This PTM involves the covalent attachment of one or more ubiquitin (Ub) proteins to a Lys (K) residue within specific target proteins through a stepwise cascade involving three enzymes: E1 (Ub-activating), E2 (Ub-conjugating), and E3 (Ub-ligase) (Vierstra, 2009). Ubiquitination confers different fates to the ubiquitinated protein depending on the number and location of the attached Ub molecules (Sadowski et al., 2012). Polyubiquitination *via* K48 Ub linkage is the best characterized form of ubiquitination and results in selective protein degradation through the ubiquitin-proteasome system (UPS) (Vierstra, 2009; Sadanandom et al., 2012).

The importance of the UPS in plants is exemplified by the fact that approximately 6% of the anticipated gene loci of the *Arabidopsis thaliana* genome (about 1600 genes) is involved in UPS-related functions (Mazzucotelli et al., 2006; Vierstra, 2009). Most of them encode putative E3 Ub-ligases that determine substrate specificity and thus are the most studied components of the ubiquitination cascade (Callis, 2014). E3 Ub-ligases can be classified into four main subfamilies [Homologous to E6-associated protein Carboxyl Terminus (HECT), Really Interesting New Gene (RING), U-box, and Cullin-RING ligases (CRLs)], depending on their subunit composition and mode of action (Vierstra, 2009). RING and U-box proteins are able to directly transfer Ub from Ub-E2 intermediates to the target (Stone, 2005; Yee and Goring, 2009). RING-type E3 Ub-ligases can also act as part of a multi-subunit CRL complex such as the SCF (Skp1, Cullin, F-box) complex in which the Cullin (CUL) protein acts as a molecular scaffold bringing together the RING protein (that binds to the E2) and the F-box protein (that directs substrate recognition) (Hua and Vierstra, 2011). In other CRL complexes, the CUL protein binds to “Bric a brac, Tramtrack and Broad Complex” (BTB) or UV-Damaged DNA-Binding Protein1 (DDB1) domain proteins that act as substrate-specific adaptors or scaffold proteins, respectively (Mazzucotelli et al., 2006). The UPS and, more particularly, E3 Ub-ligases have been shown to play a crucial role in the regulation of many steps of plant immune signaling (Marino et al., 2012). Indeed, ubiquitination modulates perception of pathogen-associated molecular patterns (PAMPs) by plasma membrane-associated receptors during PAMP-triggered immunity (PTI) and regulates the accumulation of intracellular immune receptors to prevent the constitutive activation of effector-triggered immunity (ETI), which is often associated with hypersensitive cell death (HR) at the sites of infection (Furlan et al., 2012; Li et al., 2014). In addition, E3 Ub-ligases are also involved in the regulation of signaling responses downstream of pathogen perception through, for instance, targeting vesicle trafficking components or nuclear proteins, such as transcription factors (TFs) (Zhou and Zeng, 2016).

Plant ability to survive abiotic stresses, including salinity, drought, cold, heat, radiation, and nutrient availability also relies on proteome plasticity. The UPS plays a central role in the efficient perception of these environmental stresses, as well as in the downstream responses, by suppressing stress signaling pathways during favorable growth conditions, eliminating negative regulators of signaling responses to stimulus or attenuating the signaling pathway once the stress has ceased (Lyzenga and Stone, 2012; Stone, 2014).

Beyond the importance of ubiquitination in controlling cytosolic proteins, the regulation of the nuclear machinery *via* the UPS is emerging as a crucial component of plant responses to biotic and abiotic stresses. Here we review the control of the homeostasis of nuclear proteins, especially the TFs, by the UPS, and its impact on plant adaptation to environmental signals. Due to space limitations, we focus on E3 Ub-ligases for which the nuclear target protein has been identified (**Table 1**). Finally, although hormones play a major role in the regulation of plant responses to environmental signals, we refer the reader to various reviews covering the extensively characterized role of SCF complexes in the transcriptional control of hormone signaling (Trujillo and Shirasu, 2010; Robert-Seilaniantz et al., 2011; Nagels Durand et al., 2016; Nguyen et al., 2016; Yang et al., 2016) and discuss here examples of non SCF-related hormone responses.

**Table 1:** Plant E3-Ub ligases with known nuclear targets and their function.

E3 Ub-ligase	Substrate	Function	Species	Reference
PUB10 (U-BOX)	MYC2	JA responses	*Arabidopsis thaliana*	Jung et al., 2015
MIEL1 (RING)	MYB30, MYB96	Defense responses/HR, ABA responses	*Arabidopsis thaliana Arabidopsis thaliana*	Marino et al., 2013; Lee and Seo, 2016
BOI1 (RING)	BOS1	Defense responses	*Arabidopsis thaliana*	Luo et al., 2010
EIRP1 (RING)	VpWRK11	Defense and JA responses	*Vitis pseudoreticulata*	Yu et al., 2013
HOS1 (RING)	ICE1	Cold stress	*Arabidopsis thaliana*	Dong et al., 2006
COP1 (CUL4-DDB1)	HY5	Drought stress	*Arabidopsis thaliana*	Catalá et al., 2011
DRIP1/2 (RING), CUL3-BPM (BTB)	DREB2A	Drought stress, Drought and heat stress	*Arabidopsis thaliana*	Morimoto et al., 2013; Qin et al., 2008; Morimoto et al., 2017
OsHCI1	Nuclear proteins	Thermotolerance	*Oryza sativa*	Lim et al., 2013
RGLG1/2, (RING)	ERF53	Drought stress	*Arabidopsis thaliana*	Cheng et al., 2012
AIP2, OsDSG1 (RING)	ABI3	ABA responses, Drought and salt stress	*Arabidopsis thaliana Oryza sativa*	Zhang et al., 2005; Park et al., 2010; Ning et al., 2011
CUL4-DDB1-DWA1/2, KEG (RING), ABD1 (CUL4-DDB1)	ABI5	Salt and drought stress, ABA responses	*Arabidopsis thaliana*	Lee et al., 2012; Stone et al., 2006; Seo et al., 2014
CUL3-BPM3 (BTB)	ATHB6	Salt and drought stress, ABA responses	*Arabidopsis thaliana*	Lechner et al., 2011
CUL3- NPR4	NPR1	SAR responses	*Arabidopsis thaliana*	Spoel et al., 2009
SCF	SNC1, RPS2	ETI	*Arabidopsis thaliana*	Li et al., 2010; Kim et al., 2010
CPR1 (F-BOX)	SNC1, RPS2	ETI	*Arabidopsis thaliana*	Cheng et al., 2011; Gou et al., 2011
CUL3-POB1 (BTB)	PUB17	Basal defense/HR	*Arabidopsis thaliana*	Orosa et al., 2017
SAP9 (A20/AN1)	Rad23d?	Drought stress, Cold stress, Salt stress, Defense responses	*Arabidopsis thaliana*	Kang et al., 2017
OsDIS1 (RING)	OsNEK6	Drought stress	*Oryza sativa*	Ning et al., 2011

## The UPS Mediates TF Degradation During Biotic Interactions

Extensive research has uncovered TFs as major nuclear proteins targeted by UPS activity during plant–pathogen interactions. The TF MYC2 acts as a master regulator that integrates multiple signals to coordinate plant defense and development through repression and activation of jasmonic acid (JA)/ethylene (ET)- and JA/wound-responsive gene expression, respectively (Abe, 2002; Boter, 2004; Lorenzo, 2004). The E3 Ub-ligase PUB10 interacts with and ubiquitinates MYC2 in the nucleus, targeting MYC2 for proteasomal degradation (Jung et al., 2015). The importance of this regulatory mode has been underlined in a recent report showing that polyubiquitinated MYC2 is deubiquitinated by nuclear UBIQUITIN PROTEASE12 (UBP12) and UBP13, thus counteracting PUB10 activity (Jeong et al., 2017). In agreement, roots of *pub10* mutant seedlings and those of seedlings overexpressing MYC2, UBP12, or UBP13 displayed a similar phenotype and were hypersensitive to methyl-jasmonate, whereas seedlings overexpressing PUB10 and *myc2, ubp12*, or *ubp13* mutant seedlings were hyposensitive (Jung et al., 2015). The role of PUB10, UBP12, or UBP13 in the MYC2-mediated plant response to pathogens was not tested in these reports. However, given that (i) MYC2 represses defense-related JA/ET-responsive genes and that (ii) UBP12 and UBP13 negatively regulate plant immunity (Ewan et al., 2011), it is tempting to speculate that these UPS-related proteins additionally modulate MYC2-dependent defense responses.

We previously identified the RING-type E3 Ub-ligase MIEL1 as being able to interact with the Arabidopsis defense-activating TF MYB30. This interaction leads to MYB30 proteasomal degradation and thus to the attenuation of Arabidopsis defense and HR responses (Marino et al., 2013). MYB30 positively regulates plant resistance by promoting the biosynthesis of very long chain fatty acids (VLCFAs). Coherently, *miel1* mutant plants displayed enhanced VLCFA-related gene expression and enhanced resistance responses after inoculation with bacteria (Marino et al., 2013). *MIEL1* expression is rapidly downregulated after bacterial infection, which allows MYB30 accumulation thus promoting defense and HR (Marino et al., 2013).

The RING-type E3 Ub-ligase BOI1 interacts with the Arabidopsis defense-related MYB TF BOS1 in yeast and *in planta* (Luo et al., 2010). BOI1 ubiquitinates BOS1 *in vitro* and the proteasome-dependent degradation of BOS1 was shown in Arabidopsis (Luo et al., 2010). In agreement, co-regulation of the expression of *BOI1* and *BOS1* in response to fungal treatment or cell death-promoting conditions suggests a functional link between the two proteins. However, no evidence that BOI1 targets BOS1 for proteasomal degradation *in vivo* has been reported and both *bos1* mutant and BOI1 RNAi Arabidopsis plants (in which the BOS1 protein should accumulate) display enhanced susceptibility to fungal infection (Mengiste, 2003; Luo et al., 2010). Thus, the molecular details behind the BOS1-BOI1 association remain unclear.

The RING-type E3 Ub-ligase EIRP1 promotes defense in Chinese wild grapevine (*Vitis pseudoreticulata*) by interacting with the nuclear TF VpWRKY11. VpWRKY11 activates the expression of JA-responsive genes that negatively regulate resistance to fungal infection (Yu et al., 2013). Thus, EIRP1-mediated proteasomal degradation of VpWRKY11 results in reduced JA-responsive signaling and enhanced resistance to the fungus (Yu et al., 2013).

## Regulation of Abiotic Stress-Related TFs by the UPS

In addition to the regulatory roles during plant–pathogen interactions, the UPS, and more particularly E3 Ub-ligases, play a prevalent role in the control of TF levels during plant responses to adverse environmental conditions. Plant responses to cold stress rely on the activation of CBF proteins and their downstream genes by the TF ICE1. The RING-type E3 Ub-ligase HOS1 interacts with ICE1 in the nucleus and triggers its ubiquitination and degradation. In agreement with HOS1 being a negative regulator of freezing tolerance, *HOS1* overexpression in Arabidopsis increases sensitivity to freezing stress (Dong et al., 2006).

The Arabidopsis TF HY5 regulates crucial processes including photomorphogenesis, responses to hormones and freezing tolerance (Lau and Deng, 2010; Catalá et al., 2011). Low temperatures induce *HY5* expression independently of CBF TFs, and the HY5 protein is stabilized through nuclear depletion of the RING E3 Ub-ligase COP1 within a CUL4-DDB1 complex, which results in anthocyanin accumulation and cold acclimation (Catalá et al., 2011). Interestingly, the UPS additionally controls HY5 accumulation through its interaction with the small prefolding protein PFD4 that accumulates in the nucleus in response to low temperature in a process that requires DELLA proteins (Perea-Resa et al., 2017). PFD4 attenuates anthocyanin biosynthesis during cold acclimation by promoting COP1-independent HY5 ubiquitination and proteasomal degradation (Perea-Resa et al., 2017).

The rice RING-type E3 Ub-ligase-encoding *OsHCI1* gene is induced by heat and cold treatments. Overexpression of OsHCI1 in Arabidopsis confers enhanced heat tolerance (Lim et al., 2013). Under high temperatures, OsHCI1 moves along the cytoskeleton from Golgi vesicles to the nucleus where it induces translocation of substrate nuclear proteins to the cytoplasm by monoubiquitination (Lim et al., 2013). This work highlights the importance of nucleo-cytoplasmic partitioning of E3 Ub-ligases in the regulation of plant stress responses. The TF DREB2A has been characterized as a positive regulator of drought-responsive genes by controlling the expression of water deficit-inducible genes (Sakuma, 2006; Sakuma et al., 2006). The RING E3 Ub-ligases DRIP1 and DRIP2 interact with DREB2A in the nucleus and mediate its ubiquitination and degradation *via* the UPS. In response to dehydration, Arabidopsis *drip1drip2* double mutant plants present significantly higher survival rates, compared to wild-type plants under drought conditions (Qin et al., 2008; Morimoto et al., 2013). It was thus proposed that DRIP1/DRIP2-mediated DREB2A ubiquitination and degradation occurs under normal growth conditions. Under stress, proteasomal degradation of DREB2A would be blocked leading to its accumulation and the activation of the expression of drought-responsive genes (Qin et al., 2008). A recent report identified a 30-amino acid domain in DREB2A that mediates its nuclear interaction with BTB-MATH proteins (BPMs). BPMs are substrate-recruiting components of CUL3-based E3 Ub-ligases that induce DREB2A degradation and modulate drought and heat stress responses through regulation of DREB2A stability (Morimoto et al., 2017).

An additional Arabidopsis TF involved in the regulation of drought responses is ERF53, whose expression is induced by drought. ERF53 interacts with the RING E3 Ub-ligases RGLG1 and RGLG2. RGLG2 localizes at the plasma membrane but under drought stress translocates to the nucleus, where it interacts with ERF53 to mediate its degradation. In agreement with RGLG2 being a negative regulator of drought stress responses, the *rglg1rglg2* double mutant exhibits drought tolerance (Cheng et al., 2012). These results show that, as DRIP1/2, RGLG1/2 negatively regulate drought stress responses in Arabidopsis.

The hormone abscisic acid (ABA) plays a key role in plant adaptation to abiotic stress by modulating physiological processes, such as seed dormancy and germination, seedling growth and stomatal aperture (Lopez-Molina et al., 2001; Finkelstein et al., 2005). Ubiquitination through CRLs, RING-type and U-box E3 Ub-ligases has been shown to regulate ABA signaling (Yu et al., 2015; Yang et al., 2016). The RING E3 Ub-ligase AIP2 negatively regulates the TF ABI3, a central regulator of ABA signaling (Zhang et al., 2005). In rice, OsDSG1 has been characterized as an AIP2 ortholog. Mutations in *Osdsg1* increase rice tolerance to drought and high salinity (Park et al., 2010; Ning et al., 2011). AFP, an ABI5-interacting protein, attenuates ABA signaling by facilitating ABI5 proteasomal degradation in nuclear bodies (Lopez-Molina, 2003) and the 26S proteasome subunit RPN10 targets ABI5 for degradation (Smalle et al., 2003). In addition, the RING E3 Ub-ligase KEEP ON GOING (KEG) negatively regulates ABI5 accumulation, confirming the direct regulation of ABI5 by the UPS (Stone et al., 2006). In the presence of ABA, induction of *ABI5* expression and KEG degradation result in increased ABI5 levels. Besides RING proteins, CRL-type E3 Ub-ligases also contribute to ABA signaling during abiotic stress responses. For example, CUL4-DDB1-based E3 Ub-ligases utilize DWA1 and DWA2 as substrate receptors and act as negative regulators of ABI5 abundance in the nucleus (Lee et al., 2012). In agreement, *dwa1, dwa2*, and *cul4* mutants are ABA- and salt-hypersensitive and display enhanced salt-, drought-, and ABA-responsive gene expression after ABA treatment (Lee et al., 2012). ABD1 is another CUL4-DDB1-associated protein able to interact with ABI5 in the nucleus and leading to its degradation (Seo et al., 2014). Thus, *abd1* mutants display ABA hypersensitivity, reduced stomatal aperture and water loss and, thus, improved tolerance to drought stress. In addition, the DDA1 protein is part of the substrate adaptor module of CUL4-DDB1-based E3 Ub-ligases and interacts with the ABA receptor PYL8 in the nucleus leading to its degradation (Irigoyen et al., 2014). Finally, the BTB-MATH protein BPM3 downregulates the abundance of the TF ATHB6 that negatively regulates drought responses in Arabidopsis (Söderman et al., 1999; Himmelbach et al., 2002; Lechner et al., 2011). As previously shown for ABI5 (Lopez-Molina et al., 2001), ABA negatively regulates ATHB6 protein turnover. ATHB6 overexpression and BPM silencing results in ABA insensitivity, enhanced water loss, increased transpiration and larger stomatal aperture, which correlates with attenuated ABA-dependent induction of salt-, drought-, and ABA-responsive genes (Lechner et al., 2011).

Besides its role in the control of plant defense, the RING-type E3 Ub-ligase MIEL1 regulates abiotic stresses in a ABA-dependent manner by promoting the degradation of the TF MYB96 (Lee and Seo, 2016). In the absence of ABA, MIEL1 accumulates and targets MYB96 to attenuate ABA signal transduction whereas, in the presence of the hormone, MIEL1 is degraded by the UPS, promoting MYB96 accumulation. These finding suggest that MIEL1 may facilitate the ABA-mediated crosstalk between biotic and abiotic stresses (Lee and Luan, 2012).

## Targeting of Other Nuclear Proteins by the UPS

Besides TFs, the UPS can regulate the proteolysis of additional crucial nuclear regulatory components. One example is the transcriptional co-activator NPR1, a central node for basal and salicylic acid (SA)-mediated systemic acquired resistance (SAR) through induction of *PR1* gene expression (Kinkema, 2000). NPR1 accumulation is tightly regulated by both its subcellular localization and nuclear PTMs (Kinkema, 2000; Withers and Dong, 2016). Under resting conditions, NPR1 forms multimers in the cytoplasm and is constantly degraded through its interaction with CUL3-NPR4 (Spoel et al., 2009). Pathogen-induced accumulation of SA induces a redox change that promotes dissociation of the NPR1 complex and translocation of NPR1 monomers to the nucleus, where they promote gene transcription by binding to TGA and NIMIN TFs (Zhou et al., 2000; Weigel, 2005) and by directly activating the expression of WRKY TFs (Wang et al., 2010). A recent report uncovered that a dynamic balance among different PTMs not only facilitates NPR1 degradation but also switches NPR1 association with WRKY transcriptional repressors to TGA transcriptional activators (Saleh et al., 2016). In resting conditions, NPR1 is phosphorylated at Ser55/Ser59, inhibiting its SUMOylation and promoting its interaction with WRKY70, which results in repressed *PR1* transcription (Saleh et al., 2016). After pathogen challenge, SA accumulation promotes Ser55/Ser59 dephosphorylation, NPR1 SUMOylation and dissociation from WRKY70. SUMOylation of NPR1 is required for (i) phosphorylation at Ser11/Ser15, triggering a signal amplification loop that generates more activated NPR1 able to interact with TGA TFs and induce *PR1* gene expression, and for (ii) ubiquitination by the CUL3-NPR4 E3 Ub-ligase, ensuring a strong but transient SAR response (Spoel et al., 2009; Saleh et al., 2016). Since NPR1 not only activates SAR but also suppresses ETI responses, NPR1 degradation may promote ETI by releasing its repression of HR in cells with an elevated SA concentration (Withers and Dong, 2016). Interestingly, the presence of a BTB domain in NPR1 suggests that it may act as an adaptor in a CUL3-based complex, although no NPR1 substrate has been described to date. In agreement with its role as a master regulator of SAR, the *Pseudomonas syringae* type III effector AvrPtoB has been recently shown to target NPR1 and mediate its proteasomal degradation following SA accumulation (Chen et al., 2017). This interaction, which is likely to occur in the cytoplasm prior to NPR1 nuclear translocation, results in suppressed SA-mediated defense signaling. The U-Box E3 Ub-ligase PUB17 has been described as a positive regulator of plant immunity (Yang et al., 2006; He et al., 2015), which represents a rare example of physical interaction among E3 Ub-ligases in plants. The BTB domain E3 Ub-ligase POB1 interacts with PUB17 in the nucleus promoting PUB17 proteasomal degradation (Orosa et al., 2017). As a result, POB1 acts as a suppressor of basal defense and HR responses and this requires POB1 dimerization, nuclear localization and interaction with PUB17. In addition of targeting proteins for degradation, E3 Ub-ligases can control protein turnover by modification of UPS-related proteins. SAP9 is an A20/AN1-type zinc finger protein involved in development and in the regulation of multiple stress responses in Arabidopsis. *SAP9* expression is induced in response to drought, cold, and salt stress as well as after ABA and pathogen challenge (Kang et al., 2017). SAP9 displays E3 Ub-ligase activity and interacts with Rad23d, a shuttle factor for the transport of ubiquitinated substrates to the proteasome (Kang et al., 2017). SAP9 may thus facilitate the degradation of ubiquitinated targets by promoting their interaction with Rad23. Alternatively, monoubiquitination of Ub-binding domain proteins by SAP9 could impair their ability to bind polyubiquitinated targets (Kang et al., 2017), in which case SAP9 would promote protein stability rather than degradation *via* the UPS.

In rice, RING E3 Ub-ligase OsDIS1 is mostly localized in the nucleus and negatively regulates drought stress responses both transcriptionally and post-translationally (Ning et al., 2011). OsDIS1, whose expression is induced under drought conditions, suppresses the expression of a wide range of drought-responsive genes. This could be due to OsDIS1-mediated ubiquitination of drought-related TFs, which would result in their degradation or structural modification (Ning et al., 2011). Additionally, OsDIS1 interacts with OsNEK6, a tubulin complex-related serine/threonine kinase, leading to its degradation *via* the UPS. Since OsNEK6 overexpression leads to increased tolerance to drought stress, it is possible that OSNEK6 promotes OSDIS1 translocation from the nucleus to the tubulin complex, providing an elegant example of regulation of the regulator.

## Conclusion and Perspectives

Regulation of gene expression plays a central role during plant adaptation to environmental stimuli. The UPS and more particularly E3 Ub-ligase proteins have emerged as crucial components in the control of gene expression during plant responses to biotic and abiotic signals. Here, we have mainly focused on the proteolytic activities of ubiquitination during the modulation of nuclear protein levels in response to stress. Although less studied, ubiquitination also displays non-proteolytic functions during transcription, for example in regulating chromatin dynamics by H2B histone monoubiquitination. Indeed, the RING E3 Ub-ligase HISTONE MONOUBIQUITINATION1 (HUB1) monoubiquitinates histone H2B, positively regulates resistance against necrotrophic fungi in an ET- and SA-dependent manner (Dhawan et al., 2009) and, together with HUB2, confers tolerance to salt stress (Zhou et al., 2017). Further work on non-proteolytic roles of ubiquitination should provide a more complete picture of the different functions of this important PTM.

Fine-tuning of gene expression is essential to establish an optimal response while preventing the detrimental effects caused by stress conditions. Temporal and spatial control of gene expression is achieved by the orchestrated action of diverse regulatory mechanisms that modulate the intensity and duration of signaling and ensure cellular homeostasis. Since ubiquitination is a reversible PTM, it is crucial to gain a deeper knowledge on the dynamics of ubiquitination and on its coordination with other events that control gene expression during plant adaptation to environmental stresses.

## Author Contributions

All authors listed have made a substantial, direct and intellectual contribution to the work, and approved it for publication.

## Conflict of Interest Statement

The authors declare that the research was conducted in the absence of any commercial or financial relationships that could be construed as a potential conflict of interest.
